# Serum antioxidant vitamin concentrations and oxidative stress markers associated with symptoms and severity of premenstrual syndrome: a prospective cohort study

**DOI:** 10.1186/s12905-021-01187-7

**Published:** 2021-02-02

**Authors:** Robyn A. Frankel, Kara A. Michels, Keewan Kim, Daniel L. Kuhr, Ukpebo R. Omosigho, Jean Wactawski-Wende, Lindsay Levine, Neil J. Perkins, Sunni L. Mumford

**Affiliations:** 1 Epidemiology Branch, Division of Intramural Population Health Research, Eunice Kennedy Shriver National Institute of Child Health and Human Development, 6710B Rockledge Dr. MSC 7004, Bethesda, MD 20892 USA; 2 Department of Obstetrics and Gynecology, University Hospitals Cleveland Medical Center, Case Western Reserve University, Cleveland, OH USA; 3 Department of Epidemiology and Environmental Health, School of Public Health and Health Professions, University at Buffalo, Buffalo, NY USA

**Keywords:** Antioxidants, F2-isoprostane, Oxidative stress, Premenstrual women, Vitamin A/C/E

## Abstract

**Background:**

It has been suggested that premenstrual syndrome (PMS) may derive from either elevated oxidative stress or reduced antioxidant vitamin levels in the body; however, these relationships have been minimally studied in a large cohort of healthy women. Our objective was to estimate the association between serum concentrations of antioxidant vitamins (A, C, and E) and markers of oxidative stress (F2-isoprostane) with symptoms and severity of PMS.

**Methods:**

The BioCycle study was a prospective cohort study following 259 healthy premenopausal women aged 18–44 years for up to 2 menstrual cycles. Frequency/severity of 20 PMS symptoms were assessed via questionnaires 4 times/cycle, and antioxidant vitamins and oxidative stress biomarkers were measured up to 8 times/cycle to correspond with specific cycle phases. Generalized linear models were used to estimate associations between mean antioxidant concentrations and oxidative stress biomarkers with PMS symptoms and severity; linear mixed models were used to evaluate associations with symptom severity scores within groups (e.g. depression, cravings, pain).

**Results:**

Higher concentrations of serum antioxidant vitamins were largely not associated with prevalence or severity of PMS symptoms. Though a few associations were observed, only associations between mean γ-tocopherol and decreased odds of swelling of the hands/feet survived adjustment for multiple comparisons (OR 0.33, 95% CI 0.16, 0.65, per ug/dL). However, F2-isoprostanes were associated with prevalence and severity of several symptoms specifically related to depression and cravings (depression score β** = **0.07, 95% CI 0.02, 0.12, per 10 ug/dL; cravings score β = 0.16, 95% CI 0.10, 0.22, per 10 ug/dL), as well as with classification of PMS severity (OR 1.07, 95% CI 1.01, 1.14, per 10 pg/dL), with these associations surviving adjustment for false discovery rate.

**Conclusions:**

F2-isoprostanes, but not antioxidant vitamins, were associated with select PMS symptoms, as well as symptom and severity categories. Specific symptom relationships merit further research.

## Background

Premenstrual syndrome (PMS) is a disorder of reproductive-aged women characterized by psychological, physical, and behavioral symptoms, which manifest in the days prior to the onset of menses and resolve a few days after [1–3]. Most women experience at least one PMS symptom, with 3–8% of women describing one or more as being severe [4, 5]. A variety of symptoms may be indicative of PMS, including: fluid retention, cravings, anxiety, depression, breast tenderness, fatigue, abdominal pain, and mood swings [3, 5–7]. Due to the multifaceted and sometimes severe nature of PMS, many women seek medical care or other therapies for symptom alleviation, and the burden of both the symptoms and the care seeking may be substantial [2]. This burden not only includes direct medical costs, but also extends to decreased quality of life, reduced work productivity, and for some, increased absenteeism from work or school—which may contribute to a larger economic impact [5, 8].

The biological mechanisms underlying the variation in both symptom type and severity across women are poorly understood, but changes in oxidative stress that occur before the onset of menses may play a role [9]. Literature suggests that a balance between free radicals and antioxidants is integral in maintaining immune function and overall health [10, 11] and potentially menstrual function and reproductive health as well [12]. The hypothesis that free radical-induced oxidative stress causes PMS is driven by evidence that excess reactive oxygen species (ROS) can lead to cell damage and death, which are implicated in a range of reproductive health conditions such as preeclampsia, endometriosis, and recurrent pregnancy loss [12–15]. Studies examining the presence of oxidative stress levels in women with PMS are scarce and ultimately reach conflicting conclusions [9, 16, 17]. However, recent research reports some evidence of efficacy in supplementation with other vitamins (such as magnesium [18] and vitamin B6 [19]) or herbals [20–24] as they may alter the hormonal, anti-inflammatory, or oxidative stress profile of women experiencing PMS and improve their symptoms. Women affected by PMS symptoms have been shown to be more likely to seek out many of the above forms of complementary and alternative medicine, including herbals, vitamins, and supplements to help cope with symptoms, [25] and even prefer these over traditional medical or surgical interventions [22]. This suggests that altering nutrient levels may be a relatively simple palliative for a complex gynecologic condition. As such, the etiologic contribution of oxidative stress to PMS and the potential for use of antioxidant therapy to manage associated symptoms warrants further investigation [12].

Popular media outlets promote increased antioxidant consumption as a treatment or cure for PMS symptoms without extensive research to support these recommendations. Therefore, research is needed to first determine if oxidative stress and antioxidant levels are associated with PMS symptoms before dietary changes or vitamin supplementation are recommended. In this study, we investigated associations between serum antioxidant vitamin levels and a biomarker of oxidative stress with symptoms of PMS among healthy, regularly menstruating women who were not taking supplements.

## Methods

### Participants and study design

The BioCycle Study (2005–2007) was a prospective cohort study designed to evaluate the relationship between reproductive hormones and oxidative stress levels throughout the menstrual cycle [26, 27]. The participant cohort consisted of 259 regularly menstruating, healthy women between the ages of 18–44 years, recruited from western New York. Participants were recruited in a variety of ways, including: advertising in clinical practices and the University at Buffalo student health center, paid advertising in print media, radio and television interviews, notices sent via list serves, and flyers at the university and throughout the region. For those interested, an initial screening phone call was conducted, followed by a mailing and an in-person visit. Exclusion criteria included factors that may interfere with a normal menstrual cycle or vitamin levels, such as: use of oral contraceptives during the study period or in the previous three months; use of Depo provera, implant or IUD in previous twelve months; current use of vitamin, mineral, or herbal supplements; use of prescription medications; pregnancy or breast feeding in the previous six months; reported attempts to conceive in the previous six months; diagnosis of uterine abnormalities or chronic conditions, such as ovulatory disorders and premenstrual dysphoric disorder (PMDD); and a self-reported body mass index (BMI) of < 18 or > 35 kg/m^2^ at screening [27]. Those eligible and interested after the screening visit were scheduled for a baseline enrollment visit 1–2 weeks prior to the start of their next menses. Women were followed for one (n = 9) or two (n = 250) menstrual cycles (Additional file 1: Figure S1).

Women completed up to 8 clinic visits per cycle for up to 2 cycles. Study visits were scheduled using fertility monitors (Clearblue Easy Fertility Monitor™; Inverness Medical, Waltham, Massachusetts) to coincide with critical phases of the menstrual cycle, including menstruation; mid- and late follicular phases; luteinizing hormone (LH) surge (predicted ovulation); and early, mid-, and late luteal phases [28]. At each of these visits, fasting blood samples were collected from participants from which the antioxidant vitamins and oxidative stress concentrations were measured. Participants were highly compliant with the study protocol; 94% of the women completed 7 or 8 clinic visits, and 100% completed at least 5 clinic visits per cycle.

The University at Buffalo Health Sciences Institutional Review Board (IRB) approved this study and served as the IRB designated by the National Institutes of Health under a reliance agreement. All participants provided written informed consent. Further details of the study design are described elsewhere [27].

### Antioxidant vitamin and F2-isoprostane concentrations assessment

Ascorbic acid (vitamin C), retinol (vitamin A), and α- and γ- tocopherol (vitamin E) were measured in all blood samples taken from participants across a menstrual cycle and subsequently averaged to reflect the mean levels across a cycle. Total ascorbic acid was determined by the dinitrophenylhydrazine (DNPH) method. Samples for ascorbate analysis were stabilized immediately following phlebotomy and centrifugation by adding 0.5 mL of heparin plasma to 2.0 mL of 6% meta-phosphoric acid and centrifuging at 3000×*g* for 10 min. Clear supernatant was decanted and frozen at − 80 °C for analysis. The absorbance of each DNPH derivatized sample was determined at 520 nm on a Shimadzu 160U spectrophotometer (Shimadzu Scientific Instruments, Inc.). Across the study period, the coefficient of variation (CV) for this test reported by the laboratory was 10%.

Fat-soluble vitamins (including retinol, and vitamin E components: α- and γ-tocopherol) were measured at the Kaleida Health Center (Buffalo, New York) simultaneously in serum using high performance liquid chromatography with photodiode array detection [29]. δ-tocopherol was also detected but was below the lower limit of quantification for our assay (0.28). The limits of detection were 0.0054 for retinol, 0.0768 for α-tocopherol, and 0.1052 for γ-tocopherol. The CV for these tests across the study period were < 6% for retinol and < 2% for α- and γ-tocopherol. Continuous monitoring of standard reference material 968c from the National Institute of Standards and Technology (NIST) and participation in the NIST Micronutrients Measurement Quality Assurance Program provided external checks on analytical accuracy.

Mean concentrations of antioxidants, including vitamin A, vitamin C, α-tocopherol, and γ-tocopherol, were calculated per cycle and were used in all analyses. Overall median concentrations were also compared with levels reported previously by reproductive aged women (i.e., 20–39 years) in the 2012 National Health and Nutrition Examination Survey (NHANES) to assess the comparability of our results with those of a nationally representative population [30].

Plasma free F_2_-isoprostane, a breakdown product of ROS and a marker of oxidative stress, was measured with a gas chromatography-mass spectrometry–based method by the Molecular Epidemiology and Biomarker Research Laboratory (University of Minnesota, Minneapolis, Minnesota) (CV = 9.4%).

### PMS symptom severity assessment

Frequency and severity of 20 premenstrual symptoms was assessed through questionnaires completed at four time points of each menstrual cycle: menses, follicular phase, peri-ovulation, and luteal phase (Additional file 2: Figure S2). Participants recalled the occurrence and severity of symptoms in the prior week. The symptoms included in this assessment were: sadness, crying spells, anger, nervousness, insomnia, tension, abdominal bloating, cravings of chocolates, cravings of sweets, cravings of salty foods, cravings of other foods, breast tenderness, lower abdominal cramping, general aches, backache, headache, acne outbreaks, change in appetite, fatigue, and swelling of the hands/feet. The severity of each symptom was ranked by the participant on a scale of 0 to 3 (0 = none, 1 = mild, 2 = moderate, 3 = severe). The symptoms included in this questionnaire were adapted from validated surveys—including the Daily Record of Severity of Problems (DRSP) and the Premenstrual Symptoms Screening Tool (PSST)—but slightly modified, given that DRSP and the PSST were designed to identify patients with PMDD specifically (a population excluded in our study) [31–34].

We categorized severity as none/mild (reference group) or moderate/severe to estimate odds of having a moderate or severe symptom during the premenstrual week. We then calculated severity scores for groups of related symptoms by summing the severity score of symptoms (as reported in the premenstrual week) within each grouping to generate an overall score. The groupings were established based on clinical expertise and included: depression (sadness, crying spells, anger) and anxiety (nervousness, insomnia, tension); hydration (abdominal bloating) and cravings (chocolate cravings, sweets cravings, salty food cravings, other food cravings); pain (breast tenderness, lower abdominal cramping, general aches, backache, headache); and other (acne outbreak, change in appetite, fatigue, swelling of hands or feet).

Overall PMS severity was evaluated using four different approaches, which utilize information on all symptoms from the luteal and follicular phase questionnaires from each cycle: (1) 5 or more moderate or severe symptoms during the luteal phase; (2) 8 or more moderate or severe symptoms during the luteal phase; (3) 3 or more moderate or severe symptoms where the luteal phase score was 30% greater than the follicular phase and at least one symptom was psychological (referred to as PMS-1 in the tables and results); and (4) 5 or more moderate or severe symptoms where the luteal phase score was 30% greater than the follicular phase and at least one symptom was psychological (referred to as PMS-2 in the tables and results) [8, 35, 36]. When summing the number of moderate or severe symptoms for each cycle, each of the individual cravings symptoms were combined into a single variable. These criteria were based upon various defintions of PMS—including those of the National Institute of Mental Health, [37] the American College of Obstetrics and Gynecology, [38] the American Psychiatric Association, [39] and the International Society for Premenstrual disorders (ISPMD) [40]—which were further expanded and implemented in studies such as Gollenberg et al. [35] and Borenstein et al. [8] Of note, these approaches attempt to establish the necessary temporality between pre- and post-menstrual symptoms in line with traditional PMS definitions and diagnoses that assume resolution of symptoms within 1–2 days of the onset of menses.

### Covariate assessment

At study enrollment, a trained research assistant measured height and weight for the calculation of BMI using standardized protocols. Demographics such as age, race, education, smoking habits, reproductive history, and physical activity were also collected at baseline through self-reported questionnaires. Physical activity was assessed at baseline using the International Physical Activity Questionnaire, and estimated for high, moderate, and low levels of activity based upon accepted cutoffs [41]. Dietary information was obtained using 24-h recalls (up to 4 times per cycle) and analyzed using the Nutrition Data System for Research software (version 2005) developed by the Nutrition Coordinating Center of the University of Minnesota (Minneapolis, Minnesota). Cycle-averaged measures of total energy (kcal/day) and fiber (g/day) were used in these analyses, as we previously found these intakes do not vary significantly across the cycle [42]. All covariates assessed had at least a 95% response rate.

### Statistical analysis

Demographic characteristics were compared between those with < 5 versus ≥ 5 moderate or severe symptoms during the luteal phase of either study menstrual cycle, and between those with < 8 versus ≥ 8 moderate or severe luteal phase symptoms in either cycle. Repeated measures ANOVA and McNemar’s tests were used for comparisons.

We estimated associations between mean antioxidant concentrations and F2-isoprostane concentrations from each menstrual cycle and odds of reporting a moderate/severe symptom during the premenstrual week for each cycle using generalized linear models. Next, we evaluated associations between antioxidant concentrations, F2-isoprostane concentrations, and scores for symptom severity within groups during the premenstrual week (e.g., depression, cravings, pain) using linear mixed models. We used generalized linear models to assess the association between mean antioxidant concentrations, F2-isoprostane concentrations, and overall PMS severity in each cycle using the four different classifications of PMS severity (5 or more moderate or severe symptoms, 8 or more moderate or severe symptoms, PMS-1 criterion, PMS-2 criterion). All models were adjusted for age, race, BMI, physical activity, smoking status, alcohol use, pain reliever use, and average total energy intake per cycle and accounted for repeated measures (i.e., multiple cycles per woman). Results were adjusted for multiple comparisons using the false discovery rate (FDR). An alpha of ≤ 0.05 was considered statistically significant. As antioxidants and oxidative stress measures have been shown to vary somewhat over the menstrual cycle [26, 43], we also evaluated associations between time-varying measures of antioxidants and oxidative stress, with time-varying symptoms as a sensitivity analysis. Splines were used to evaluate the assumption of linearity. We did not find evidence to suggest that linear modeling was inappropriate (e.g., quadratic or restricted cubic spline modeling did not help explain the associations in our population). All statistical analyses were calculated using SAS 9.4 (SAS Institute, Cary, North Carolina).

## Results

Five or more moderate or severe symptoms were reported in 34% of cycles, and 8 or more were reported in 14% of cycles (Table 1). Most demographic characteristics did not differ between women based on their number of moderate or severe symptoms per cycle. However, women who did not report severe symptoms were more likely to have completed up to a high school or lesser level of education as well as report no alcohol intake in the past 12 months. A trend towards higher BMI among those with 5 or more severe symptoms versus those reporting less was also observed.

**Table 1:** Demographic characteristics of women in the BioCycle Study by symptom severity classification per cycle

	Total	Total with data on PMS symptoms^a^	5 or more moderate or severe symptoms		*p* value	8 or more moderate or severe symptoms		*p* value
			Yes	No		Yes	No	
Number of cycles [*n* (%)]	509	490	167 (34)	323 (66)		67 (14)	423 (86)	
Age (years) ^b^	27.4 ± 8.2	27.3 ± 8.2	27.1 ± 8.2	27.4 ± 8.2	0.67	28.2 ± 8.7	27.2 ± 8.1	0.32
Race [*n* (%)]								
White	302 (59)	290 (59)	99 (59)	191 (59)	0.12	34 (51)	256 (61)	0.15
Black	101 (20)	97 (20)	26 (16)	71 (22)		13 (19)	84 (20)	
Other	106 (21)	103 (21)	42(25)	60 (19)		20 (30)	83 (20)	
≤ High School Education [*n* (%)]	65 (13)	62 (13)	14 (8)	48 (15)	0.04	2 (3)	60 (14)	0.01
Current Smoker [*n* (%)]	20 (4)	19 (4)	9 (5)	10 (3)	0.22	3 (5)	16 (4)	0.73
Physical activity [*n* (%)]								
Low	48 (9)	47 (10)	13 (8)	34 (11)	0.61	5 (8)	42 (10)	0.80
Moderate	182 (36)	174 (36)	62 (37)	112 (35)		23 (34)	151 (36)	
High	279 (55)	269 (55)	92 (55)	177 (55)		39 (58)	230 (54)	
BMI (kg/m^2^)	24.1 ± 3.9	24.1 ± 3.9	24.5 ± 3.8	23.9 ± 3.9	0.07	24.9 ± 3.6	24.0 ± 3.9	0.06
Nulliparous [*n* (%)]	367 (74)	356 (74)	119 (74)	237 (74)	0.94	47 (70)	309 (75)	0.45
Total calories (kcal)	1608 ± 405	1608 ± 407	1611 ± 413	1607 ± 404	0.92	1622 ± 415	1606 ± 406	0.76
Fiber (g/day)	13.6 ± 6.0	13.5 ± 5.7	13.7 ± 5.3	13.4 ± 5.8	0.55	14.3 ± 5.4	13.4 ± 5.7	0.19
Currently sexually active [*n* (%)]	267 (71)	255 (70)	99 (74)	156 (67)	0.16	40 (73)	215 (69)	0.75
Ever sexually active [*n* (%)]	378 (75)	365 (75)	133 (82)	232 (72)	0.03	55 (82)	310 (74)	0.22
Alcohol (in the past 12 months)								
None	172 (34)	165 (34)	55 (33)	110 (34)	0.04	19 (28)	146 (35)	0.0007
1 drink	54 (11)	51 (10)	18 (11)	33 (10)		11 (16)	40 (9)	
2 drinks	144 (28)	137 (28)	47 (28)	90 (28)		16 (24)	121 (29)	
3 drinks	80 (16)	78 (16)	20 (12)	58 (18)		9 (13)	69 (16)	
4 drinks	38 (7)	38 (8)	13 (8)	25 (8)		2 (3)	36 (9)	
≥ 5 drinks	21 (4)	21 (4)	14 (8)	7 (2)		10 (15)	11 (3)	
Use of pain relievers	143 (28)	134 (28)	54 (33)	80 (25)	0.09	23 (34.3)	111 (26.5)	0.19

^a^19 cycles with missing information on PMS symptoms

^b^Values are mean ± standard deviation and n (%) as indicated

The most frequently reported symptoms included lower abdominal cramping (42% of cycles), abdominal bloating (38%), tension (33%), and breast tenderness (28%) (Table 2). Food cravings were also frequent (sweets 22%; chocolate 24%; salty foods 14%; other 10%). Thirty-one percent of cycles met the PMS-1 criterion and 24% met the PMS-2 criterion.

**Table 2:** Associations between mean serum antioxidant and F2-isoprostane concentrations and presence of any moderate or severe PMS symptoms per cycle by symptom type

	# Cycles with moderate/severe symptoms (%)	Vitamin A (ug/dL)	Vitamin C (ug/dL)	α-tocopherol (ug/dL)	γ-tocopherol (ug/dL)	F2-isoprostane (pg/mL)
		OR (95% CI), per ug/dL	OR (95% CI), per ug/dL	OR (95% CI), per ug/dL	OR (95% CI), per ug/dL	OR (95% CI), per 10 pg/dL
Depression						
Sadness	72 (14)	0.43 (0.02, 10.71)	1.12 (0.69, 1.83)	1.03 (0.91, 1.16)	0.86 (0.51, 1.46)	1.07 (0.99, 1.16)
Crying spells	42 (8)	0.02 (0.00004, 11.30)	0.70 (0.34, 1.42)	0.99 (0.82, 1.19)	0.97 (0.49, 1.91)	**1.11 (1.02, 1.20)**^**a**^
Anger	116 (23)	0.30 (0.01, 9.38)	0.98 (0.63, 1.52)	0.93 (0.83, 1.04)	0.91 (0.60, 1.39)	**1.17 (1.05, 1.29)**^**a**^
Anxiety						
Nervousness	103 (21)	0.32 (0.01, 9.50)	1.26 (0.77, 2.07)	1.00 (0.89, 1.12)	0.76 (0.50, 1.15)	1.02 (0.91, 1.14)
Insomnia	33 (7)	8.72 (0.10, 796.01)	2.11 (0.90, 4.91)	1.19 (1.003, 1.41)^a^	0.97 (0.49, 1.89)	0.92 (0.73, 1.16)
Tension	165 (33)	0.25 (0.01, 4.96)	0.70 (0.46, 1.08)	0.92 (0.84, 1.02)	0.89 (0.63, 1.26)	**1.11 (1.03, 1.19)**^**a**^
Hydration						
Abdominal bloating	188 (38)	0.05 (0.003, 0.93)^a^	0.96 (0.61, 1.52)	0.92 (0.84, 1.02)	0.77 (0.52, 1.15)	1.01 (0.95, 1.06)
Cravings						
Chocolate	119 (24)	0.08 (0.004, 1.57)	0.81 (0.49, 1.32)	1.03 (0.91, 1.16)	0.82 (0.55, 1.24)	**1.10 (1.02, 1.18)**^**a**^
Sweets	112 (22)	0.26 (0.01, 6.01)	0.85 (0.53, 1.39)	1.05 (0.97, 1.13)	0.79 (0.52, 1.19)	1.08 (0.99, 1.19)
Salty	72 (14)	12.63 (0.39, 409.03)	1.05 (0.60, 1.85)	0.98 (0.87, 1.11)	1.28 (0.73, 2.22)	1.07 (1.01, 1.13)^a^
Other food	49 (10)	0.13 (0.001, 15.18)	0.78 (0.39, 1.52)	1.01 (0.90, 1.14)	0.82 (0.50, 1.36)	**1.32 (1.12, 1.57)**^**a**^
Pain						
Breast tenderness	139 (28)	0.10 (0.004, 2.5)	0.76 (0.41, 1.38)	0.98 (0.90, 1.08)	0.88 (0.57, 1.34)	0.90 (0.79, 1.04)
Lower abdominal cramping	209 (42)	0.06 (0.005, 0.90)^a^	1.04 (0.70, 1.56)	0.98 (0.89, 1.07)	0.72 (0.51, 1.01)	1.06 (0.95, 1.18)
General aches	89 (18)	0.35 (0.01, 13.04)	1.06 (0.53, 2.09)	0.98 (0.85, 1.12)	0.65 (0.40, 1.07)	1.00 (0.94, 1.06)
Backache	119 (24)	0.15 (0.005, 4.62)	1.02 (0.55, 1.89)	0.93 (0.81, 1.07)	0.77 (0.49, 1.21)	1.01 (0.96, 1.07)
Headache	97 (19)	2.33 (0.11, 49.33)	1.04 (0.61, 1.77)	1.00 (0.92, 1.09)	1.04 (0.67, 1.61)	1.04 (0.96, 1.13)
Other						
Acne outbreak	85 (17)	0.15 (0.005, 6.60)	1.37 (0.76, 2.49)	0.96 (0.84, 1.10)	0.64 (0.39, 1.04)	1.09 (1.003, 1.18)^a^
Change in appetite	97 (19)	0.02 (0.0004, 1.26)	1.06 (0.61, 1.83)	0.97 (0.87, 1.09)	0.70 (0.45, 1.09)	1.08 (1.01, 1.16)^a^
Fatigue	122 (24)	0.34 (0.01, 8.19)	1.12 (0.69, 1.83)	0.99 (0.89, 1.10)	0.81 (0.55, 1.21)	1.03 (0.96, 1.10)
Swelling of hands/feet	32 (6)	0.0008 (0.000001, 0.5)^a^	1.38 (0.66, 2.89)	0.92 (0.75, 1.13)	**0.33 (0.16, 0.65)**^**a**^	0.94 (0.72, 1.21)

Adjusted for energy intake, age, BMI, race, physical activity, smoking, alcohol intake, and pain reliever use. Bold indicates statistically significant after adjusting for multiple comparisons using the False Discovery Rate

^a^Indicates statistical significance at the 0.05 level

Average concentrations for antioxidant vitamins A, C, and E among the BioCycle participants were comparable with those of the NHANES population (whose participants shared similar demographics with our participants) in that all serum vitamin values fell within normal limits; ascorbic acid concentrations were slightly higher, while retinol, α-tocopherol, and γ-tocopherol were lower among women in the BioCycle Study (ascorbic acid, umol/L [normal range: 35.37–176.84 umol/L]: BioCycle median 96, NHANES median 55; retinol, ug/dL [normal range: 32.5–78.0 ug/dL]: BioCycle median 37, NHANES median 49; α-tocopherol, ug/dL [normal range: 550–1700 ug/dL]: BioCycle median 796, NHANES median 997; γ-tocopherol, ug/dL [normal range < 430 ug/dL]: BioCycle median 173, NHANES median 196) [30].

Antioxidant vitamin and F2-isoprostane concentrations were associated with select moderate/severe PMS symptoms during the premenstrual week (Table 2). Higher mean vitamin A concentrations were associated with decreased abdominal bloating (OR 0.05, 95% CI 0, 0.93; per ug/dL), lower abdominal cramping (OR 0.06, 95% CI 0.005, 0.90; per ug/dL), and swelling of hands/feet (OR 0.0008, 95% CI 0.000001, 0.5; per ug/dL); higher mean α-tocopherol concentrations were associated with increased odds of insomnia (OR 1.19, 95% CI 1.003, 1.41; per ug/dL); and higher mean γ-tocopherol concentrations were associated with reduced odds of moderate/severe swelling or the hands or feet (OR 0.33, 95% CI 0.16, 0.65; per ug/dL). F2-isoprostane concentrations were positively associated with reporting of moderate/severe crying spells (OR 1.11, 95% CI 1.02, 1.20; per 10 pg/mL), anger (OR 1.17, 95% CI 1.05, 1.29; per 10 pg/mL), and tension (OR 1.11, 95% CI 1.03, 1.19; per 10 pg/mL), as well as change in appetite (OR 1.08, 95% CI 1.01, 1.16; per 10 pg/mL) and chocolate (OR 1.10, 95% CI 1.02, 1.18; per 10 pg/mL), salty (OR 1.07, 95% CI 1.01, 1.13; per 10 pg/mL), and other food cravings (OR 1.32, 95% CI 1.12, 1.57; per 10 pg/mL), and acne (OR 1.09, 95% CI 1.00, 1.18; per 10 pg/mL) (Table 2). After adjustment for false discovery rate, associations between γ-tocopherol and swelling of hands or feet, as well as between F2-isoprostanes and crying spells, anger, tension, chocolate cravings, and other food cravings remained significant.

Antioxidant vitamin concentrations were not associated with symptom severity scores within the symptom groupings, though F2-isoprostane concentrations were associated with higher depression and craving symptom scores after adjustment for false discovery rate (depression score β** = **0.07, 95% CI 0.02, 0.12, per 10 ug/dL; cravings score β = 0.16, 95% CI 0.10, 0.22, per 10 ug/dL, Table 3). F2-isoprostane concentrations were also associated with increased odds of PMS symptom severity using the various PMS classifications outlined **(**PMS-1 OR 1.07, 95% CI 1.01, 1.14, per 10 pg/mL; PMS-2 OR 1.09, 95% CI 1.02, 1.17, per 10 pg/mL; Table 4).

**Table 3:** Associations between mean serum antioxidant concentrations and F2-isoprostane (per 1 unit increase) and symptom scores by cycle

Symptoms score	Vitamin A (ug/dL)	Vitamin C (ug/dL)	α-tocopherol (ug/dL)	γ-tocopherol (ug/dL)	F2-isoprostane (pg/mL)
	β (95% CI), per ug/dL	β (95% CI), per ug/dL	β (95% CI), per ug/dL	β (95% CI), per ug/dL	β (95% CI), Per 10 pg/dL
Depression	− 1.92 (− 4.19, 0.34)	− 0.03 (− 0.39, 0.33)	− 0.05 (− 0.13, 0.03)	− 0.15 (− 0.46, 0.16)	**0.07 (0.02, 0.12)**^**a**^
Anxiety	− 0.72 (− 2.97, 1.53)	0.08 (− 0.25, 0.41)	0.01 (− 0.07, 0.09)	− 0.14 (− 0.42, 0.15)	0.05 (0.003, 0.10)
Fluid retention/hydration	− 1.02 (− 2.12, 0.08)	0.02 (− 0.15, 0.20)	− 0.01 (− 0.04, 0.02)	− 0.11 (− 0.25, 0.04)	0.01 (− 0.01, 0.03)
Craving	− 2.33 (− 6.69, 2.03)	− 0.08 (− 0.72, 0.57)	0.004 (− 0.15, 0.16)	− 0.33 (− 0.88, 0.23)	**0.16 (0.10, 0.22)**^**a**^
Pain	− 3.18 (− 7.03, 0.68)	− 0.06 (− 0.77, 0.65)	− 0.05 (− 0.19, 0.08)	− 0.45 (− 0.94, 0.04)	0.05 (− 0.06, 0.16)
Other	− 1.55 (− 3.59, 0.49)	0.31 (0.001, 0.63)^a^	− 0.01 (− 0.08, 0.06)	− 0.24 (− 0.50, 0.01)	0.03 (− 0.01, 0.07)

Adjusted for energy intake, age, BMI, race, physical activity, smoking, alcohol intake, and pain reliever use. Bold indicates statistically significant after adjusting for multiple comparisons using the False Discovery Rate

^a^Indicates statistical significance at the 0.05 level

**Table 4:** Associations between mean serum antioxidant and F2-isoprostane concentrations (per 1 unit increase) and odds of 5 or more or 8 or more moderate or severe PMS symptoms or PMS per cycle

	# cycles with PMS (% of total cycles)	Vitamin A (ug/dL)	Vitamin C (ug/dL)	α-tocopherol (ug/dL)	γ-tocopherol (ug/dL)	F2-isoprostane (pg/mL)
		OR (95% CI), per ug/dL	OR (95% CI), per ug/dL	OR (95% CI), per ug/dL	OR (95% CI), per ug/dL	OR (95% CI), Per 10 pg/dL
5+ moderate or severe symptoms	167 (34)	0.02 (0.001, 0.56)^a^	1.20 (0.75, 1.92)	0.99 (0.90, 1.09)	0.80 (0.54, 1.19)	1.10 (1.02, 1.19)^a^
8+ moderate or severe symptoms	67 (14)	0.04 (0.0004, 3.61)	1.06 (0.62, 1.81)	1.02 (0.90, 1.15)	0.47 (0.27, 0.85)^a^	1.08 (0.99, 1.17)
PMS-1^a^	148 (31)	0.27 (0.01, 5.57)	0.81 (0.53, 1.25)	0.92 (0.83, 1.03)	0.78 (0.52, 1.15)	1.07 (1.01, 1.14)^a^
PMS-2^b^	111 (24)	0.07 (0.003, 1.93)	1.18 (0.77, 1.81)	1.01 (0.90, 1.13)	0.82 (0.53, 1.27)	1.09 (1.02, 1.17)^a^

Adjusted for energy intake, age, BMI, race, physical activity, smoking, alcohol, and pain reliever use

*PMS* premenstrual syndrome

^a^PMS-1: Participant reported 3 or more moderate or severe symptoms, where at least one was psychological and the luteal phase score was 30% greater than the follicular phase

^b^PMS-2: Participant reported 5 or more moderate or severe symptoms, where at least one was psychological and the luteal phase score was 30% greater than the follicular phase

In a sensitivity analysis evaluating time-varying antioxidant vitamin and F2-isoprostane concentrations with time-varying PMS symptoms, we observed largely similar findings between F2-isoprostanes and depression related symptoms (Additional files 3 and 4: Tables S1 and S2). Associations between γ-tocopherol and lower symptom severity scores for depression, fluid retention, pain, and other symptoms were also observed.

## Discussion

Among a cohort of healthy regularly menstruating women not taking dietary supplements, serum concentrations of antioxidant vitamins A, C, and E were generally not associated with PMS symptoms or severity. However, F2-isoprostanes—a biomarker of oxidative stress—showed associations with multiple psychological and craving symptoms, as well as with classification of PMS. These findings suggest that, at levels characteristic of the US population, oxidative stress may influence the development of specific PMS symptoms and contribute to disease severity, however the evidence to support the use of antioxidant vitamins as a remedy for the syndrome in its entirety is inconclusive.

Prior research suggested that an imbalance between ROS and antioxidant vitamins and the subsequent increase in oxidative stress could negatively influence ovarian and menstrual cycle functioning, potentially contributing to PMS symptom onset [9, 17]. As a result, an increase in dietary or supplemental antioxidant vitamins A, C, and E has been proposed both by epidemiologic research and popular informational outlets as a potential therapy to reverse the onset of oxidative stress. While the use of many antioxidant vitamins has been studied as treatment for other oxidative stress-induced pathologies, few studies to our knowledge have explored the efficacy of antioxidant vitamins in reducing PMS symptoms amongst a large population of women.

Support for the use of antioxidants in treating the diverse symptoms associated with the menstrual cycle stems from literature demonstrating its relationship with a multitude of other physical and psychological diseases. For example, several studies indicate that treatment with vitamin E and C supplementation may reduce oxidative stress mediated inflammation associated with conditions like nonalcoholic steatohepatitis [44–46]. Oxidative stress has also been implicated in the progression of Alzheimer’s disease and other neurodegenerative disorders; both animal and human studies exploring vitamins C and E therapies found significant delay in cognitive decline in those with these conditions [47, 48]. Vitamin E supplementation also contributed to the reduced risk of morbidity in patients with diabetes mellitus and associated higher levels oxidative stress levels [49]. Even those with major depressive disorder have been shown to have dysregulated oxidative stress levels [50, 51]. Though the exact mechanisms are not well understood, we hypothesized that oxidative stress and antioxidants may similarly influence a complex physical and psychological disease process like PMS.

We identified few studies exploring a relationship between biomarkers of oxidative stress and PMS symptoms, with weak support for the use of antioxidants as therapy. In a small study, Duvan and colleagues found increased levels of lipid hydroperoxide (another biomarker of oxidative stress) and decreased total antioxidant levels before menstruation relative to after among women with PMS (n = 20) [9]. They did not, however, identify differences in other biomarkers of oxidative stress (malondialdehyde [MDA], protein carbonyl) or antioxidant status (total thiol) across the menstrual cycle or between women experiencing and not experiencing PMS [9]. However, Kalia et al. [17] found that MDA was higher after menstruation among their control (n = 6) versus PMS (n = 6) groups, and Balat et al. [16] found nitric oxide was higher after menstruation relative to before among women experiencing PMS (n = 11). In all of these studies, small population sizes likely limit statistical power and generalizability.

The largest study that examined oxidative stress and PMS that we identified (n = 40 women with PMS and n = 40 without PMS) found higher concentrations of oxidative stress markers among women with PMS, but did not detect statistically significant changes across the cycle within their study groups [52]. A few older and smaller studies generally observed no significant deficiencies in antioxidant vitamins among women with PMS [53–55]. The existing research may indicate the potential for oxidative stress to influence PMS, but we took a much-needed step to evaluate associations between several antioxidants, a measure of oxidative stress, and multiple symptoms linked to this syndrome within a larger population of women contributing data from multiple menstrual cycles.

The positive associations between F2-isoprostane on psychological symptoms such as crying, anger, and tension could be explained by an underlying disturbance in the GABAergic neuroendocrine system by ROS. Alterations in these neurotransmitter levels by ROS can lead to neuronal cell damage, causing changes in mood and behavior and the development of symptoms associated with depression and anxiety [56]. This may be interrelated to the positive relationships between F2-isoprostane and food cravings, as the depressive symptoms caused by ROS have been shown to cause increased appetite and obesity [57]. The relationship between vitamin A and abdominal bloating could possibly be explained by its role in maintaining gut microbial and immunologic homeostasis [58, 59]. The relationship between higher γ-tocopherol levels and reduced swelling has not been specifically studied in prior research, and further research is needed to understand potential mechanisms.

Currently, the only supplements recommended by the American College of Obstetricians and Gynecologists for PMS symptoms are calcium (to reduce both physical discomforts and mood symptoms) and magnesium (to help reduce water retention, breast tenderness, and mood symptoms) [60]. The utility of antioxidant compounds, such as vitamins A, C, and E, which can be obtained from fruits, vegetables or dietary supplements [61], has been studied in the context of other gynecologic and reproductive conditions [62, 63]. A randomized clinical trial showed significant reduction in PMS symptoms with administration of 400 IU of α-tocopherol compared to placebo, with the greatest improvements seen in social impairment and sexual drive among women suffering severe PMS [64]. Some research supports associations between other vitamin intakes and symptoms reduction. A case–control study using women from the Nurse Health Study II found an inverse relationship between intake of thiamine (B1) and riboflavin (B2) from food sources and incidence of PMS; however, B vitamin supplementation was not associated with a lower risk of PMS [65]. Overall, there is limited evidence to support use of antioxidant dietary supplements to improve PMS symptoms [66].

Our study had many strengths. We assessed associations between multiple vitamins and PMS symptoms among a large cohort of healthy, regularly menstruating women not taking supplements. Although we utilized self-reported symptoms rather than medical diagnoses, most PMS in the general population is not formally or methodically diagnosed by a health practitioner and as such, our evaluation is reflective of actual PMS experiences. Formal diagnosis of PMS by a practitioner typically requires 2 months of prospective symptom evaluation, which parallels and authenticates the length of time of this study period [6]. Importantly, we were able to assess individual symptoms as well as composite measures of PMS; in identifying associations with the former, we indicate that relying on composite measures of PMS with symptoms unique to each woman or study population may yield comparisons across studies that are etiologically uninformative or difficult to interpret. We also evaluated serum concentrations of antioxidant vitamins, as opposed to dietary intake, which is a more precise method for assessing these exposures, as they take into account the variability in individual metabolism [67].

Our study also had some limitations. The study population was fairly homogenous, and enrollment centered on healthy women. Since participants reporting PMDD, a severe form of PMS, were excluded from this study, we may have been unable to estimate associations with antioxidant concentrations that become apparent with more severe symptoms. As this was an analysis of secondary outcomes within the BioCycle Study, no a priori power calculations were conducted; we recognize that study power may limit our ability to detect very modest associations between antioxidant biomarkers and PMS symptoms. As we only followed women for 2 cycles, we were also unable to look at long-term changes in antioxidant concentrations or oxidative stress associated with changes in PMS severity. Additionally, while this study evaluated an array of PMS symptoms, it did not assess their potential impact on quality or life or daily functioning. Further research is needed to evaluate this complex, multifaceted relationship.

## Conclusion

In conclusion, these data do not support the hypothesis that serum antioxidant vitamin concentrations are associated with the presence or severity of PMS symptoms among healthy, regularly menstruating women. However, our data support the idea that oxidative stress may influence certain types of PMS symptoms, specifically symptoms related to depression and cravings, as well as disease severity. Other researchers exploring antioxidant levels or therapies in the context of PMS should be wary of grouping symptoms or creating scores as this may skew potential associations. Our study provides a foundation for future mechanistic research on the relationship between these antioxidants and oxidative stress and specific symptoms, as well as the precise role and targeted therapy for oxidative stress in PMS symptoms.

## Supplementary Information

**Additional file 1: Figure S1.** Recruitment flow chart for women participating in the BioCycle Study. Figure adapted from Wactawski-Wende et al. (2009).

**Additional file 2: Figure S2.** PMS Symptoms Questionnaire. Excerpt from the Clinical Visit Questionnaire Assessing PMS Symptoms. This questionnaire was administered 4 times per cycle during menses, early follicular phase, expected ovulation, and the mid-luteal phase.

**Additional file 3: Table S1.** Associations between time-varying serum antioxidant and F2-isoprostane concentrations and presence of any moderate or severe PMS symptoms per cycle phase by symptom type.

**Additional file 4: Table S2.** Associations between time-varying serum antioxidant and F2-isoprostane concentrations and symptom scores by cycle.

## Data Availability

The datasets used and/or analyzed during the current study will be made available from the corresponding author upon request.

## Abbreviations

BMI: Body mass index
CV: Coefficient of variation
FDR: False discovery rate
IRB: Institutional Review Board
LH: Luteinizing hormone
NHANES: National Health and Nutrition Examination Survey
NIST: National Institute of Standards and Technology
PMDD: Premenstrual dysphoric disorder
PMS: Premenstrual syndrome
ROS: Reactive oxygen species
