# Supplementary material for: Serum antioxidant vitamin concentrations and oxidative stress markers associated with symptoms and severity of premenstrual syndrome: a prospective cohort study
Source: BMC Womens Health. 2021 Feb 2;21:49. doi: 10.1186/s12905-021-01187-7 (PMC7851915; doi:10.1186/s12905-021-01187-7)
Supplement: Supplementary file 1 — Additional file 1: Figure S1. Recruitment flow chart for women participating in the BioCycle Study. Figure adapted from Wactawski-Wende et al. (2009). [file 12905_2021_1187_MOESM1_ESM.doc]

Supplementary Figure 1. Recruitment flow chart for women participating in the BioCycle Study. Figure adapted from Wactawski-Wende et al. 2009 (20).

957 calls received

449 screening visits done

484 not interested or self-screened out

24 recruitment ended before able to join

318 eligible

276 enrolled

42 not enrolled

131 ineligible

9 completed

1 cycle

250 completed

2 cycles

17 withdrew before completing 1 cycle

259 women

509 total cycles were evaluated

490 cycles had PMS symptom quality and severity data available
