# Supplementary material for: Serum antioxidant vitamin concentrations and oxidative stress markers associated with symptoms and severity of premenstrual syndrome: a prospective cohort study
Source: BMC Womens Health. 2021 Feb 2;21:49. doi: 10.1186/s12905-021-01187-7 (PMC7851915; doi:10.1186/s12905-021-01187-7)
Supplement: Supplementary file 2 — Additional file 2: Figure S2. PMS Symptoms Questionnaire. Excerpt from the Clinical Visit Questionnaire Assessing PMS Symptoms. This questionnaire was administered 4 times per cycle during menses, early follicular phase, expected ovulation, and the mid-luteal phase. [file 12905_2021_1187_MOESM2_ESM.doc]

Supplemental Figure 2: Excerpt from the Clinical Visit Questionnaire Assessing PMS Symptoms. This questionnaire was administered 4 times per cycle during menses, early follicular phase, expected ovulation, and the mid-luteal phase.

The following questions are designed to evaluate signs and symptoms associated with premenstrual syndrome and difficult menstruation. Please enter a check mark in the box most closely describing the severity of your experience with **EACH** of the following symptoms, from none to severe over the **PAST 7 DAYS.** Please be sure to provide an entry for each of the listed symptoms leaving no question blank, even if you are not premenstrual or menstruating.

|  | **Symptom** | **None** | **Mild** | **Moderate** | **Severe** |
| --- | --- | --- | --- | --- | --- |
| a. | Depression or sadness | ① | ② | ③ | ④ |
| b. | Tension or irritability | ① | ② | ③ | ④ |
| c. | Anxiety or nervousness | ① | ② | ③ | ④ |
| d. | Anger, aggression, short temper | ① | ② | ③ | ④ |
| e. | Crying spells | ① | ② | ③ | ④ |
| f. | Swelling of hands or feet | ① | ② | ③ | ④ |
| g. | Breast tenderness or fullness | ① | ② | ③ | ④ |
| h. | Abdominal bloating | ① | ② | ③ | ④ |
| i. | Lower abdominal cramping | ① | ② | ③ | ④ |
| j. | Generalized aches and pains | ① | ② | ③ | ④ |
| k. | Lower backache | ① | ② | ③ | ④ |
| l. | Headache | ① | ② | ③ | ④ |
| m. | Fatigue | ① | ② | ③ | ④ |
| n. | Change in appetite | ① | ② | ③ | ④ |
| o. | Craving for chocolate | ① | ② | ③ | ④ |
| p. | Craving for sweets in general | ① | ② | ③ | ④ |
| q. | Craving for salty flavor | ① | ② | ③ | ④ |
| r. | Other food cravings | ① | ② | ③ | ④ |
| s. | Insomnia | ① | ② | ③ | ④ |
| t | Acne or outbreak of pimples or blemishes | ① | ② | ③ | ④ |
