# Supplementary material for: Serum antioxidant vitamin concentrations and oxidative stress markers associated with symptoms and severity of premenstrual syndrome: a prospective cohort study
Source: BMC Womens Health. 2021 Feb 2;21:49. doi: 10.1186/s12905-021-01187-7 (PMC7851915; doi:10.1186/s12905-021-01187-7)
Supplement: Supplementary file 3 — Additional file 3: Table S1. Associations between time-varying serum antioxidant and F2-isoprostane concentrations and presence of any moderate or severe PMS symptoms per cycle phase by symptom type. [file 12905_2021_1187_MOESM3_ESM.docx]

Supplemental Table 1: Associations between time-varying serum antioxidant and F2-isoprostane concentrations and presence of any moderate or severe PMS symptoms per cycle phase by symptom type^1^

|  |  | **Vitamin A**  **ug/dL** | **Vitamin C**  **ug/dL** | **α- tocopherol**  **ug/dL** | **γ-tocopherol**  **ug/dL** | **F2-isoprostane pg/mL** |
| --- | --- | --- | --- | --- | --- | --- |
|  | **# Cycles with Moderate/ Severe symptoms (%)** | **OR (95% CI), per ug/dL** | **OR (95% CI), per ug/dL** | **OR (95% CI), per ug/dL** | **OR (95% CI), per ug/dL** | **OR (95% CI), per 10 pg/dL** |
|  |  |  |  |  |  |  |
| *Depression* |  |  |  |  |  |  |
| Sadness | 72 (14) | 0.92 (0.10, 8.12) | 0.71 (0.46, 1.08) | 0.97 (0.88, 1.08) | 0.81 (0.63, 1.04) | 1.02 (0.99, 1.04) |
| Crying spells | 42 (8) | 0.23 (0.01, 7.39) | 0.74 (0.46, 1.18) | 0.95 (0.82, 1.11) | 0.71 (0.45, 1.12) | 0.98 (0.92, 1.04) |
| Anger | 116 (23) | 1.47 (0.22, 9.87) | 0.99 (0.72, 1.36) | 0.98 (0.92, 1.06) | 1.11 (0.90, 1.37) | 1.02 (1.001, 1.05) ^2^ |
| *Anxiety* |  |  |  |  |  |  |
| Nervousness | 103 (21) | 3.79 (0.67, 21.41) | 1.01 (0.73, 1.40) | 1.00 (0.93, 1.08) | 1.02 (0.81, 1.29) | 1.00 (0.96, 1.05) |
| Insomnia | 33 (7) | 0.69 (0.04, 12.29) | 1.71 (1.11, 2.64) ^2^ | 1.09 (1.01, 1.18) ^2^ | 1.55 (1.10, 2.17) ^2^ | 1.00 (0.99, 1.02) |
| Tension | 165 (33) | 1.40 (0.23, 8.48) | 0.73 (0.53, 1.01) | 0.94 (0.87, 1.01) | 0.98 (0.80, 1.20) | **1.03 (1.01, 1.04)** ^2^ |
| *Hydration* |  |  |  |  |  |  |
| Abdominal bloating | 188 (38) | 0.08 (0.01, 0.61) ^2^ | 0.69 (0.47, 0.995) ^2^ | 0.90 (0.82, 0.99) ^2^ | 0.73 (0.57, 0.94) ^2^ | 1.01 (0.99, 1.02) |
| *Cravings* |  |  |  |  |  |  |
| Chocolate | 119 (24) | 0.10 (0.01, 1.01) | 0.76 (0.53, 1.09) | 0.97 (0.89, 1.06) | 0.82 (0.61, 1.10) | 1.00 (0.96, 1.04) |
| Sweets | 112 (22) | 0.39 (0.04, 3.81) | 0.75 (0.52, 1.08) | 0.93 (0.84, 1.03) | 0.79 (0.59, 1.06) | 0.98 (0.93, 1.04) |
| Salty | 72 (14) | 1.62 (0.16, 16.39) | 0.65 (0.39, 1.08) | 1.04 (0.97, 1.12) | 1.14 (0.81, 1.60) | 0.99 (0.95, 1.03) |
| Other food | 49 (10) | 0.52 (0.02, 11.84) | 1.45 (0.87, 2.41) | 1.02 (0.91, 1.14) | 0.62 (0.38, 1.04) | 1.00 (0.96, 1.03) |
| *Pain* |  |  |  |  |  |  |
| Breast tenderness | 139 (28) | 0.51 (0.06, 4.72) | 0.89 (0.48, 1.64) | 0.93 (0.84, 1.03) | 0.72 (0.54, 0.94) ^2^ | 1.01 (0.99, 1.03) |
| Lower abdominal cramping | 209 (42) | 0.49 (0.08, 3.25) | 0.86 (0.62, 1.21) | 0.98 (0.91, 1.06) | 0.76 (0.59, 0.96) ^2^ | 1.01 (0.99, 1.02) |
| General aches | 89 (18) | 5.24 (0.53, 51.6) | 0.91 (0.55, 1.50) | 0.93 (0.82, 1.05) | 0.80 (0.54, 1.19) | 0.98 (0.94, 1.03) |
| Backache | 119 (24) | 5.59 (0.69, 44.99) | 1.05 (0.65, 1.70) | 0.97 (0.88, 1.07) | 0.98 (0.69, 1.41) | 1.00 (0.98, 1.02) |
| Headache | 97 (19) | 2.61 (0.31, 22.03) | 1.00 (0.73, 1.37) | 1.05 (0.99, 1.12) | 0.83 (0.64, 1.08) | 0.99 (0.97, 1.02) |
| *Other* |  |  |  |  |  |  |
| Acne outbreak | 85 (17) | 1.78 (0.22, 14.09) | 1.34 (0.93, 1.93) | 0.93 (0.84, 1.03) | 0.72 (0.54, 0.95) ^2^ | 1.00 (0.99, 1.02) |
| Change in appetite | 97 (19) | 0.46 (0.05, 3.89) | 1.04 (0.74, 1.47) | 1.01 (0.94, 1.10) | 0.88 (0.66, 1.18) | 0.97 (0.87, 1.07) |
| Fatigue | 122 (24) | 0.45 (0.08, 2.60) | 1.12 (0.82, 1.52) | 1.00 (0.94, 1.06) | 0.85 (0.68, 1.06) | 0.99 (0.97, 1.02) |
| Swelling of hands/feet | 32 (6) | 0.36 (0.01, 12.47) | 1.41 (0.76, 2.63) | 0.91 (0.74, 1.12) | **0.45 (0.28, 0.74)** ^2^ | 1.02 (1.004, 1.03) ^2^ |

^1^ Adjusted for energy intake, age, BMI, race, physical activity, smoking, alcohol, and pain reliever use.

^2^ Indicates statistical significance at the 0.05 level

Bold indicates statistically significant after adjusting for multiple comparisons using the False Discovery Rate.
