# Supplementary material for: Serum antioxidant vitamin concentrations and oxidative stress markers associated with symptoms and severity of premenstrual syndrome: a prospective cohort study
Source: BMC Womens Health. 2021 Feb 2;21:49. doi: 10.1186/s12905-021-01187-7 (PMC7851915; doi:10.1186/s12905-021-01187-7)
Supplement: Supplementary file 4 — Additional file 4: Table S2. Associations between time-varying serum antioxidant and F2-isoprostane concentrations and symptom scores by cycle. [file 12905_2021_1187_MOESM4_ESM.docx]

Supplemental Table 2: Associations between time-varying serum antioxidant and F2-isoprostane concentrations and symptom scores by cycle^1^

|  | **Vitamin A**  **ug/dL** | **Vitamin C**  **ug/dL** | **α- tocopherol**  **ug/dL** | **γ-tocopherol**  **ug/dL** | **F2-isoprostane pg/mL** |
| --- | --- | --- | --- | --- | --- |
| Symptoms Score | **β (95% CI),**  **per ug/dL** | **β (95% CI),**  **per ug/dL** | **β (95% CI),**  **per ug/dL** | **β (95% CI),**  **per ug/dL** | **β (95% CI),**  **per 10 pg/dL** |
| Depression | -0.49 (-1.50, 0.53) | -0.04 (-0.19, 0.11) | -0.04 (-0.08, 0.002) | **-0.13 (-0.24, -0.01) ^2^** | **0.01 (0.004, 0.02) ^2^** |
| Anxiety | -0.17 (-1.18, 0.83) | -0.02 (-0.17, 0.14) | -0.03 (-0.07, 0.0009) | -0.03 (-0.15, 0.08) | 0.01 (-0.01, 0.02) |
| Fluid Retention/Hydration | -0.36 (-0.82, 0.09) | -0.05 (-0.12, 0.03) | -0.02 (-0.04, 0.002) | **-0.07 (-0.12, -0.01) ^2^** | -0.00004 (-0.0004, 0.004) |
| Craving | -1.29 (-2.81, 0.22) | -0.13 (-0.39, 0.13) | -0.03 (-0.10, 0.04) | -0.21 (-0.43, 0.003) | 0.002 (-0.02, 0.02) |
| Pain | -0.35 (-1.94, 1.24) | -0.15 (-0.45, 0.14) | -0.04 (-0.09, 0.02) | **-0.21 (-0.40, -0.02) ^2^** | -0.002 (-0.02, 0.02) |
| Other | 0.14 (-0.72, 1.01) | 0.11 (-0.04, 0.26) | -0.02 (-0.05, 0.02) | **-0.12 (-0.23, -0.02) ^2^** | -0.0009 (-0.01, 0.01) |

^1^Adjusted for energy intake, age, BMI, race, physical activity, smoking, alcohol, and pain reliever use

^2^ Indicates statistical significance at the 0.05 level

Bold indicates statistically significant after adjusting for multiple comparisons using the False Discovery Rate.
